# Children’s experiences of participating in a school-based health promotion parental support programme – a qualitative study

**DOI:** 10.1186/s12887-021-02694-0

**Published:** 2021-05-11

**Authors:** Mahnoush Etminan Malek, Gisela Nyberg, Liselotte Schäfer Elinder, Emma Patterson, Åsa Norman

**Affiliations:** 1 Karolinska Institutet, Department of Global Public Health, Tomtebodavägen 18 A, 171 77 Stockholm, Sweden; 2 The Swedish School of Sport and Health Sciences, Lidingövägen 1, 114 33 Stockholm, Sweden; 3 Centre for Epidemiology and Community Medicine, Region Stockholm, Solnavägen 1E, 113 65 Stockholm, Sweden

**Keywords:** Diet, Physical activity, Socioeconomic position, Elementary school, Parental involvement

## Abstract

**Background:**

Children’s voices are seldom heard in process evaluations concerning health promotion programmes. A Healthy School Start Plus (HSSP) is a parental support programme, conducted in Sweden, with the aim of promoting healthy diet, physical activity and preventing obesity in preschool class children. The 6-month programme includes: (1) Health information to parents; (2) Motivational Interviewing with parents by school nurses; (3) Classroom activities and home assignments for children; (4) A self-test of type-2 diabetes risk for parents. We aimed to describe children’s experiences of the third component regarding barriers and facilitators of participating in and learning from the classroom activities in the HSSP.

**Methods:**

In total 36 children from 7 schools in Sweden, mean age 6 years, participated in 7 focus group discussions. Purposeful sampling with maximum variation was used to collect the data. The focus groups were audio-recorded, transcribed and analysed using qualitative content analysis.

**Results:**

Four categories were identified; (1) Time available to work on intervention activities; (2) Others’ interest; (3) Abilities and interests in intervention activities; and (4) Practicing the concept of health.

**Conclusions:**

The findings may improve the HSSP and other similar interventions that include classroom-based learning regarding health by highlighting the following points to consider: aiming for homework to be an integrated part of the school-setting to enhance parental involvement; using flexible material, tailored to the children’s abilities and giving children adequate time to finish the intervention activities; and making teachers and parents aware of the importance of verbal and body language regarding intervention activities.

**Trial registration:**

The Healthy School Start Plus trial was retrospectively registered in the International Standard Randomised Controlled Trial Number Registry on January 4, 2018 and available online at ClinicalTrials.gov: No. NCT03390725.

**Supplementary Information:**

The online version contains supplementary material available at 10.1186/s12887-021-02694-0.

## Background

The continuous rise in the prevalence of overweight and obesity worldwide during the last 50 years highlights the pressing need for effective strategies for the prevention and management of weight disturbances [1, 2]. Children with obesity face social, emotional and physical challenges and if excess weight is retained into adulthood, it can lead to an increased risk for e.g. type-2 diabetes (T2D), cardiovascular diseases, certain cancers and reduced life expectancy [3–5]. The combined literature suggests that efforts to prevent overweight and obesity should be multi-component and target both physical activity and dietary behaviours. A recent systematic review indicated that interventions targeting children and adolescents aged 6 to 12 years combining both physical activity and diet may reduce the risk of obesity, without resulting in adverse effects or increasing health inequalities [6]. In addition, another review emphasised that school-based interventions combining physical activity and diet and involving parents may be an effective strategy for childhood obesity prevention worldwide, and highlighted the need for more research in non-school settings [7].

Although the rise in BMI in children and adolescents has levelled off in some high-income countries, social inequalities in overweight and obesity persist or even increase, with higher prevalence among children from families with a low socioeconomic position  (SEP) [8]. In Sweden social inequalities in health, can be partly attributed to unhealthy dietary habits, low physical activity levels and obesity, all of which are more common in adults with low SEP as well as in children from families with low SEP. There is a need for programmes for health promotion and obesity prevention, targeting socially disadvantaged areas [9–11].

The school setting is an optimal setting for health promotion initiatives and provides an opportunity to reach all children, no matter their SEP or weight status, therefore it is where most programmes take place [7]. In addition, several systematic reviews have concluded that interventions to prevent child overweight and obesity should start at an early age [6] in various school and primary health care settings and that such interventions obtain stronger effects when parents are directly involved [12–15]. However, the Swedish National Board of Health and Welfare has pointed out in a report that there is a lack of evidence-based programmes targeting health-related behaviours for use in Swedish school health care [16]. We have previously developed and trialled the school-based Healthy School Start programme [17, 18] and the Healthy School Start Plus (HSSP) intervention [19] is the third iteration.

Process evaluations are important as they complement effectiveness studies, providing knowledge about barriers and facilitators of implementation. Children’s voices are rarely heard in process evaluations of health promotion and obesity prevention programmes. The United Nations Convention on the Rights of the Child (UNCRC) article 12 emphasises that children have a right to express their views in matters concerning them, no matter how young they are [20]. In part due to UNCRC, the literature concerning how to conduct qualitative research with children is growing [21]. Interviewing children poses specific challenges as, in contrast to adults, they are more interested in the present and the concrete, rather than the abstract [22]. Nonetheless, children as young as four years old can provide insights into their health experiences in their daily lives, e.g. living with cancer, pain or chronic diseases [21]. Although there are challenges and dilemmas to consider when involving children in qualitative research, including them provides valuable knowledge about their opinions and experiences [23]. This data is otherwise unobtainable [24], and could improve the effectiveness of the programme in question.

This study aimed to describe children’s experiences of barriers and facilitators of, and learning from, the classroom lessons included in the HSSP intervention [19]. By listening to the voices of six-year-old children, valuable findings of their experiences and views will add knowledge to the existing literature, as well as provide support on how children can be involved in future process evaluations. These findings could be of great value in the future planning and scaling-up of similar school-based programmes for this age group, and will be used in future implementations of the HSSP programme.

## Methods

A qualitative design was used to explore the experiences and views of the children that took part of the programme. This design is suitable for process evaluations and to study issues in depth [25].

### Description of the programme

The overall aim of the parental support programme HSSP is to prevent unhealthy weight development through promotion of healthy dietary and physical activity behaviours, in the home setting, in pre-school class children (six-year-olds). The duration of the programme was 6 months, from November 2017 to May 2018. A detailed description of the programme can be found in the study protocol [19]. The HSSP is evaluated as a cluster-randomised controlled parallel trial, with randomisation at school level. The effectiveness of this programme has been compared to standard school routines, usual classroom activities and a health check where height and weight were measured, which is all the control groups were exposed to (to be published). The programme builds on Social Cognitive Theory (SCT) [26] and a slightly modified version has been evaluated twice before [17, 18]. Both previous studies showed beneficial results regarding diet and the second study also found a reduction in BMI z-score among children with obesity compared to the control group, however effects were short-lived [17, 18]. Based on those previous experiences, and findings from process evaluations of the programme [27–29], a refined version of the HSSP was developed. This time, the parental components had a clearer focus on parenting, the parents’ cooperation with one another and how they are important role models for their children. Moreover, in order to facilitate future integration of the programme into routine practice, all intervention components are now carried out by school staff. In addition, a fourth component targeting parents’ risk of T2D was introduced in order to increase parents’ motivation for change of health-related behaviours for the whole family, if needed. The intervention components are: (1) A health information brochure to parents concerning healthy parenting practices; (2) Motivational interviewing sessions with parents performed by the school nurse; (3) Classroom activities and a workbook with home assignments for the children; and (4) A web-based self-test of T2D risk, the FINDRISC test [30], for parents.

The focus of this study is on the classroom activities and home assignments for the children, components that involved the children directly. The classroom activities aimed to increase the children’s knowledge and to influence their attitudes and preferences towards health-related behaviours through experienced-based activities. The home assignments were intended to stimulate the parents to practice role modelling in an easy way. Nine lessons were taught to the children by teachers, who were encouraged to follow a manual when carrying out the lessons, with free adaptation of the activities. Health information materials and the workbook for the children, and instructions to teachers were available on the project’s website. The teacher’s manual contained activities and topics related to health e.g. the importance of eating healthily, trying new vegetables and fruits, the importance of being physically active, trying different movement activities and listening to their own heartbeat. The home assignments for the children to conduct with their parents were linked to the topics of the lessons, e.g. to have a weekly screen-free day, look for healthy food in the grocery store and to be physically active together as a family. The parents received instructions related to the home assignments from the teachers during the intervention, and pre-intervention from the research team at the parental kick-off meeting at the start of the school year for each school.

### Setting and participants

Schools in some of the most disadvantaged areas in and around the Stockholm region, defined by a proportion of parents with post high school education of less than 50 %, were invited to participate in the HSSP programme. At national level this proportion is 57 % [31]. Seventeen schools accepted the invitation of which eight were randomised to intervention schools with 155 participants, and nine to waiting list control schools with 197 participants.

Purposeful sampling was used to identify a sample for this study with the aim to include 30 children. Exclusion criteria were parent’s and children’s inability to express themselves in plain Swedish and other specific reasons making it difficult for their children to attend the focus groups, e.g. autism spectrum disorder. All other parents in the intervention group were eligible for inclusion and a maximum variation was applied using the following characteristics: the sex of the child, parents’ country of birth and the child’s weight status. We aimed to achieve maximal variation with regard to these parameters and to select information-rich cases related to the aim and to increase transferability [25, 32, 33]. One of the eight intervention schools was excluded from the study, as there were too few children who could express themselves in Swedish. Based on the selected characteristics of maximal variation, six to ten eligible children in the remaining seven intervention schools were identified by two of the authors (MEM and ÅN), and the teachers were approached through email and asked to help with the final sampling. After reaching consensus with the teachers six children were chosen from each school, based on the aim of achieving maximal variation as described above, and an invitation letter was mailed to the guardians of the children. The guardians were then approached by telephone and the first consent was given orally, followed by a written consent. Of all guardians approached, one declined to allow their child to participate without further explanation. The child was replaced with a child with similar characteristics. A further six invited children did not participate in the study due to illness. Each child received a gift card via the parent, worth about ten euros for participating in the study. In total 36 children aged six to seven years, participated in this study. The participants’ characteristics are summarised in Table 1.

**Table 1:** Descriptive characteristics of the participating children at baseline

	Total sample
Sociodemographic characteristics	*n* = 36
Mean age, years (SD)	6.3 (0.3)
Sex % (n)	
Girl	63.9 (23)
Boy	36.1 (13)
Weight status^a^ % (n)	
Underweight	5.6 (2)
Normalweight	66.7 (24)
Overweight	16.7 (6)
Obesity	11.0 (4)
Parental level of education^b^ % (n)	
High	69.4 (25)
Low	25.0 (9)
Missing	5.6 (2)
Parental country of birth^c^ % (n)	
Sweden/Nordic	44.4 (16)
Europe	2.8 (1)
Outside Europe	47.2 (17)
Missing	5.6 (2)

^a^Weight status is defined according to the International Obesity Task Force and based on baseline data, collected September**-**October 2017

^b^Parental level of education is based on the highest reported education level within a family, defined as low; ≤ 12 years and high; > 12 years

^c^Parental country of birth is based on the country of birth of the mother, in cases where two countries where reported

### Data collection

Focus group methodology is suitable when aiming to describe experiences and attitudes and data is generated through the interaction between the participants [34]. Seven focus groups were performed, all were audio-recorded, with four to six participants in each group during May and June 2018. An interview guide was developed, according to the format described by Krueger [34] and the content of the open ended questions were inspired by the Consolidated Framework for Implementation Research (CFIR) [35]. The domains most relevant for this study were; intervention characteristics, inner setting, outer setting and characteristics of individuals [35]. Examples of questions were: “what did you do during the lessons”, “what did you find difficult/easy”, “what did you think about it?”, “what do you think your teachers thought about it”. The questions were not pilot tested. However, they were adjusted to fit the different groups, the moderator accustomed the language used and used probing when appropriate.

The focus groups were carefully pre-planned. The interview guide can be found in the supplement (see Additional file 1). In order to gain a sense of the content of the work conducted in class, the moderator (MEM) read logs written by the teachers regarding how they had conducted the classroom lessons, what topics and activities were included. The teachers had received questions to answer concerning each lesson. Examples of questions were: “how long time did the lesson take?”, “which parts of the lesson were conducted”, “can you grade the children’s appreciation of the lesson between one to five?”, “how did the home-assignment work”. MEM further looked for signs of intervention activities in the children’s classrooms where the focus groups were conducted and used it to facilitate the discussion. During the discussion, pictures drawn, the workbook and other documents that the children had worked with during the intervention were shown. This included the Swedish plate model and the Keyhole labelling, a front-of-pack labelling system which indicates foods with less salt, sugar, fat and more fibre than other products in the same category [36]. The atmosphere was intended to be playful and friendly, and everyone sat in a circle on the floor. Prior to the start of the focus group, the moderator introduced herself and explained the reasons for doing the research. In the introduction the children were assured that there were no right or wrong answers and the focus groups started with a presentation game; presentation of name and favourite food, while holding a toy. Each focus group had a duration of 20–30 min and when the children seemed tired and unfocused, a movement activity was performed. All focus groups were conducted by the moderator and an assistant, the assistant took field notes and helped making the children feel comfortable.

### Ethics approval and consent to participate

The HSSP study was ethically approved by the Regional Ethical Review Board in Stockholm No. 2017/711 − 31/1 and conducted in accordance to the Declaration of Helsinki. Written and oral informed consent was collected from the children’s guardians. Guardians were encouraged to discuss with their children whether to participate or not and in the beginning of the focus groups the children were asked if they were informed about the purpose of the interview. This was repeated by MEM and the children had the right to cancel or withdraw from participating in the focus groups at any time. To ensure anonymity, the names of the participating children are replaced by numbers when presenting results.

### Data analysis

The data was analysed using qualitative content analysis [37] with an inductive approach according to Elo & Kyngäs [38]. The focus groups were transcribed verbatim by MEM and read several times. During the transcription, notes of nonverbal communication e.g. [pointed at the wall], were taken to allow for an extended understanding of the children’s verbal communication (by MEM). ÅN peer-reviewed the analysis conducted by MEM and read the transcripts independently. MEM is a dietitian and a PhD student within HSSP with comprehensive experience in working with children. ÅN is the project leader for HSSP and a postdoctoral researcher, an anthropologist, trained in behavioural science with extensive experience in qualitative research and family-centred health promotion. Meaning units indicating barriers and facilitators related to the research question were marked and labelled with codes (by MEM). To strengthen the credibility, independent co-coding and discussions between MEM and ÅN were conducted until consensus was reached [25]. Hereafter, inductive categories and subcategories were created (by MEM), based on similarities and differences among the codes. Discussions between MEM and ÅN were held during the creation of categories and subcategories until consensus was reached. As the data was on a manifest level, the analysis was kept on this level throughout the whole process, and no overarching theme on a latent level was derived. Notes were taken during the analysis process and quotes were highlighted. In cases of uncertainty, in the finalisation of the results MEM listened to the audio-files again to ensure the trustworthiness of the quotations. All authors discussed the categories and subcategories until consensus was reached.

To ensure anonymity, the focus groups were assigned numbers (FG 1–7) and so were the children (C1-6). In cases of simultaneous speaking, the children in question and the citations are underlined. The nonverbal communication and explanation of the context of the citations are written within square brackets [X]. The focus groups were conducted and transcribed in Swedish and translation of the selected quotes into English was performed after reaching consensus among all authors.

## Results

The analysis revealed four categories describing the children’s experiences of barriers and facilitators related to the classroom activities and home assignments. The categories were: (1) Time available to work on intervention activities; (2) Others’ interest; (3) Abilities and interests in intervention activities; and (4) Practicing the concept of health. The findings of the analysis with the categories and subcategories are presented in Table 2.

**Table 2:** Categories and subcategories with the corresponding barriers and facilitators as experienced by children

Categories			
Time available to work on intervention activities	Others’ interest	Abilities and interests in intervention activities	Practicing the concept of health
	***Subcategories***		***Subcategories***
B: Lack of time B: Being absent B: Frustrating to wait F: Getting more time	***Support and help*** B: Lack of support and help F: Received support and help F: Pedagogical support at the children's level	B: Activity or topic boring B: Activity or topic difficult F: Activity or topic fun F: Activity or topic easy	***Aids for learning*** F: Use of symbols for recognition of the health message F: Involvement in practical assignments F: Use of material that facilitates learning and discussion
	***Enthusiasm*** B: Helpers negative B: Helpers show no enthusiasm F: Helpers positive F: Helpers show enthusiasm		***Opportunities to apply knowledge in everyday life*** F: Practicing knowledge at home F: Relating knowledge to health behaviours of people in one’s surrounding F: Knowledge produces certainty in own health behaviour

B = Barriers

F = Facilitators

### Time available to work on intervention activities

This category comprises barriers and facilitators related to time for the activities in class and when working on the assignments at home. Some children described that they were not given enough time to be able to finish some activities or assignments in the workbook. In addition, when a child was absent from class due to e.g. sickness, the child did not get time to catch up on activities and then felt as if he or she was being left out of the activity. Some children also described that it was frustrating at times to wait for an activity to take place, they wanted to carry out the activity straight away. Here one child expresses disappointment when missing parts from a lesson where an activity was performed:

C1: Yes it’s because I had a doctor’s appointment and missed everything [the lesson]. Then I went back to school and only got to cut three sodas…That was no fun [to only get to do a small part of the lesson]. (FG 2).

The facilitator within this category was “Getting more time”. The children wanted to finish activities that were not completed, and they wanted more time to do so. During focus groups some children described:

[Recognizing the workbook] C1: Yes..can we finish?..Can we finish working in them? [looking through the workbook]. C2: I still have [the assignment] sleep and rest left to do. C3: Can we finish this one today? [the workbook]. (FG 5).

### Others’ interest

This category comprises of the two subcategories ‘Support and help’ and ‘Enthusiasm’.

#### Support and help

 Some children described a lack of support and help from their parents and from teachers and other people in their surroundings when working with the activities. Most children brought the workbook home to finish with their parents and a clear barrier was the lack of support and help given to the children while doing the assignments. Some children described how they did not receive any help and that they had to do the assignments all by themselves. Other children described receiving support and help while working with the activities, from their parents and the teachers in school. Some children received help from other children, e.g. siblings or cousins, in the family:

C1: Nobody. [helped me]. C2: Mom. C3: Yes on the first page it was… C4: Yes on the first page..and with the football…[I was helped by]…my big sister… C3: I don’t even have siblings. C5: Nobody. C6: Mom.… C2: Mom. C3: Nobody…I did all [the assignments] myself. (FG 2).

Some children described receiving support from the teachers in class, some through encouraging words or affirmations such as being rewarded with stars. Other children described that the teachers explained it in a way that they could understand:

C1, C2, C3: Easy [to understand what the keyhole was]. C3: First it was difficult, you did not understand, but then when [the teachers] told us it became easy… (FG 1).

#### Enthusiasm

Some children described how the teachers and the parents did not show any enthusiasm or that they were negative towards the intervention activities. In many cases this was related to the parents either expressing a negative opinion about the intervention or the home assignments, or not showing any enthusiasm about helping with the assignments, e.g. by saying that they were too tired. The children also described how the different helpers sometimes showed enthusiasm or had a positive attitude. The children’s experience of the helper’s intention to assist them with the different activities were often based on the children’s interpretation of the state of mind or body language of the teachers and the parents. When asked how their parents felt about helping them, the children described their different experiences of their parents’ engagement and attitude:

C1: Mom thought it was hard…C2: and my mom…C3: and my dad…C4: My dad didn’t think it was hard…C5: and my brother…and my dad helped me […] they thought it was good… C4: My mom thought…she was a little tired… she wanted to lie down. [C3 was asked] C3: Mom. C3: [my mother thought it was] fun…she wasn’t tired. (FG 7).

A clear facilitator within this category was the enthusiasm shown and children’s interpretation of face mimics. Signs of happiness, such as smiling, was interpreted as the helpers thinking something was good or enjoyable. Here some children discussed their perception of the teacher’s thoughts about the lessons:

C1: Maybe …well, was fun. Because, once when we talked about that, [the teacher] smiles when she talks sometimes […] mm, yes [many children]. C2: She did this [showing a smile] C3: They looked happy. I thought they looked. they were like…they thought it was good. (FG 3).

### Abilities and interests in intervention activities

This category comprises of barriers and facilitators related to children’s subjective abilities and interests in the intervention activities. The children described the different activities and topics being difficult or boring, and when they described something as being boring, like e.g. cutting small pieces with scissors or drawing, they could refer to the activity as being difficult at the same time. The children also described certain activities and topics as being easy or fun, e.g. the children could say that it was fun to do practical activities such as tasting new foods, drawing and listening to the heartbeat after performing a physical activity. Here as well, the children’s impressions of an activity as fun was meant that it was also easy to carry out. The children’s views of whether the intervention activities were difficult or easy differed greatly amongst the children:

C1:…and the candy bag was easy [discussing different activities]. The keyhole was easy…the plate model was in the middle... And then the heart was hard. […] C2: I haven’t done the heart [the assignment]. C3:.it was difficult to draw and then cut out… (FG 5).

### Practicing the concept of health

This category comprises the two subcategories ‘Aids for learning’ and ‘Knowledge applied in everyday life’.

#### Aids for learning

This subcategory only comprises facilitators. The children described how they used the intervention activities and the material to facilitate learning through focusing on and discussing the concept of health. The classroom lessons and the home assignments included graphic material such as the Swedish plate model and the Keyhole labelling, which the children could relate to. They described how they had worked with these symbols and could also link them to healthy choices. Here some children discuss:

[Recognizing the Keyhole] Yessss! [many children]. C1: Keyhole. C2: I can’t remember what it’s called, I remember what it looks like though. C3: It’s … it’s like...I think it’s some food marking or something. C4:.. that it is healthy. Like… that these are the things that are healthy, like for example many things […]. C5: I was going to say another thing...that we should remember that […]. [Recognizing the plate model] C5: Like, it is… I do not remember if we talked about it although I know that, like, how much vegetables and fruits you eat every day and then there are also some things.. like, this is the kind of food that is good to eat… (FG 3).

The intervention material and the practical assignments facilitated learning and gave the children an opportunity to discuss health. Here the children discussed the different things they had experienced and learnt:

C1: I remember talking about that it is good to move and not good to sit and have the Ipad all the time. […] The plate model. […] Ahh, we drew what we ate…uh we sat and talked, and we drew what we ate in the workbook. C2: That you become healthy by healthy stuff, yes. C1: We also talked about that you should eat fruits and vegetables every day. […] C3: If you eat too much candy you can get…have holes in your teeth. C1:.and teeth may fall off. […] C1: that it sounded very loud [listening to the heart] C2: and the pulse sounded. […] Good. [many children, how it felt]. C1: That you can feel the pulse on the neck, on the wrist and on the arm … [C4 shows how to take the pulse on the neck] C1: I did it at home [took the pulse]. C2: And [the teacher] said we could try a movement activity, so we danced and then we felt it pounding. (FG 1).

#### Opportunities to apply knowledge in everyday life

This subcategory only comprises facilitators. By working with different health tasks in different settings, the children could apply knowledge and evaluate their environment from a health perspective. The children could e.g. link the activities to their environment in relation to healthy and unhealthy behaviours. One facilitator within this subcategory was the opportunity to practice knowledge at home. The children described that they had worked with the intervention tasks in school, at home, in the shop, e.g. finding products with the keyhole, and during their vacation, e.g. having a screen-free day, and had practiced their knowledge in different settings. The children could relate to the intervention activities and the concept of health in relation to other people’s behaviours in their surroundings. Here two children describe:

C1: Mm. That like...that [the teacher] said like this…that you can eat candy like on Saturdays, but you should not eat like… today, on Tuesday I eat a bag of candy and on Monday I eat like…because sometimes...the children from fifth grade, sometimes like… they usually buy at ICA [a supermarket] and eat at school. They can buy a big bag of maybe [chocolate] every day… […] C2: That my cousin, she eats sweets every day and she never brushes her teeth… she does not brush her teeth or anything and she is six years… and because she eats sweets [so often] so she went to the dentist and pulled out her teeth and now she has two black teeth. (FG 3).

One facilitator was 'Knowledge produces certainty in own health behaviour'. Here one child described learning more about food in school as being useful in the home environment:

C1: Thumbs up [lessons about healthy eating] because you felt like, you felt like good because you like…now I know...now I can say to my mom that I don’t want to eat that much because I...then I will feel bad…because then you know like…how much you should eat. (FG 3).

## Discussion

The Healthy School Start Plus programme was carried out in schools in disadvantaged areas and focuses on parental support. This study aimed to investigate children’s experiences of barriers and facilitators related to taking part in and learning from one component of the intervention, namely the classroom activities with accompanying home assignments. Interviewing the children led to insights and added to the knowledge of children’s needs and desires, and four categories of barriers and facilitators were identified. The findings revealed that children, with their different abilities and interests, wanted adequate time to work on the intervention activities, that they received different amounts of support from their parents and people in their surroundings, and that both verbal and body language was important for how they received information. The findings also revealed that the children used the intervention activities and the material to facilitate learning, and by working with different health tasks in different settings, the children could apply knowledge and evaluate their environment from a health perspective. The findings may improve the HSSP and other similar interventions that include classroom-based learning regarding health.

### Challenging to involve all parents

The findings of this study show that the children did not always get the support they needed, and they could recall who supported and helped them, as well as a lack of support or help. Hence, this study highlights the importance of further enhancing parental involvement, an important factor for improving effects in health promotion interventions, targeting children [6]. Two process evaluations of the previous version of the HSSP have been conducted and support this. The first study showed that it is crucial for implementation success that there is a clear communication between teachers and parents, with well-defined roles [27]. The second study underlined the importance of adapting the intervention to the abilities of the target group to enhance participant engagement, and that it is important to target parents as role models as well as parents’ cooperation, and cooperation between parents and school, to be able to achieve changes in the home environment [28]. These aspects were taken into consideration during the development of HSSP and efforts were made to enhance parental involvement. The workbook given to the children to finish with their parents, a component from previous trials now enhanced by an instruction sheet for parents on how to practice positive parenting practices through each home assignment, was intended to help the parents to get involved, practice role modelling and take part of the programme together with their children, in an easy way [19]. However, despite all this, during the focus groups it was clear that some children did not receive any help from their parents to finish the assignments. As the start of preschool-class in Sweden is the first-year children attend school, many children and the parents have not yet been introduced to homework routines. This might have been the reason why some parents did not help their children with the assignments. If the classroom activities with the home assignments were more integrated in the school curriculum, and homework routines were introduced at the start of preschool-class, it might have been easier for the teachers to motivate parents to get involved in the homework. A study from England involving parents and children aged six to seven years suggested that future childhood obesity prevention programmes should allow a degree of flexibility to enable adaptation to individual school and family circumstances [39]. In line with this, when implementing HSSP, in schools where homework routines are not common in preschool-class, it may be important to establish a plan for the introduction of it, e.g. by setting up rules and expectations concerning homework assignments, and allow the schools to adapt the plan to fit their circumstances and knowledge of how to best reach the parents of their particular school. The importance of involving parents is consistently shown to be important [40]. Clarke et al. emphasised that parental involvement facilitates consistency of messages between school and home and also improves parental knowledge [39]. By engaging parents in school-based health promotion and obesity prevention programmes children can get the same message and practice healthy behaviours both in school and at home, thereby avoiding conflicting messages concerning healthy and unhealthy behaviour. Contradictory messages in different settings can create confusion for children and may hinder their ability to make healthy lifestyle choices [41]. Although efforts have been made to engage parents as much as possible in HSSP, more work is needed to understand how best to reach and involve parents, something also highlighted as an important factor for intervention effectiveness in childcare settings [42], where possible factors could include a firmer structure for home assignment and improve communication between home and school [27, 28, 39].

### The packaging and delivery of health information is important

It is important to establish what children need and want, in order to be able to increase their knowledge and improve their own health behaviour. Our findings indicate that the children had different opinions on which activities they thought were fun or boring and the level of difficulty of the different activities and assignments. Throughout the development of HSSP efforts were made to ensure the acceptability of the intervention components to fit not only the children, but also the teachers and the parents [19]. The material used was written in easy-to-read Swedish to fit all levels of parental education. Furthermore, the teachers were encouraged to tailor the lessons and the activities to the knowledge and abilities of the children in their class. Even though these efforts were made, not surprisingly, the children had different opinions. Our findings show that the use of pedagogical material, such as the practical activities, the symbols and the inclusion of a workbook for the children to take home and finish with their parents, enabled the children to get familiar with and enhance their knowledge of healthy and unhealthy behaviour. Our findings also show that children notice very subtle expressions by adults e.g. the children’s description that their teachers were smiling during a lesson was interpreted as something positive, this indicates that it may mean a lot for their experience and learning. This is in line with Social Cognitive Theory, which highlights the importance of role modelling and observational learning [26]. Hence, it seems important that teachers and parents are aware of the crucial value of both verbal and body language when talking about health-related issues with young children.

Our study suggests that children, as young as six-year-olds in the case of HSSP, with the right support and use of pedagogical material can be empowered and both learn more about healthy and unhealthy behaviours and put their knowledge into practice in their daily lives. This finding is supported by Clarke et al. who indicated that children participating actively in the intervention acted as agents of change by encouraging their parents to change their habits [39]. Moreover, a study conducted in Spain using focus groups among 8- and 12-year-old children emphasised that school pupils should be considered as health agents, who, with the support of teachers, can increase control over their own health [43]. During the focus groups it was evident that the children had enjoyed, learnt and experienced new things through the classroom activities and the home assignments. These results are also similar to those reported by Clarke et al. who reported that children and parents valued the healthy lifestyle interventions delivered through schools and that they reported changes in family lifestyle behaviour, skills and knowledge [39]. In line with this fifth-grade children participating in focus groups were unanimous in their enjoyment of taking part of the Healthy Lifestyles Programme (HeLP) that was specifically designed to encourage children to take healthy lifestyle messages home to their families [44]. The children in our study showed a willingness to carry out and finish the different assignments and requested more time to do so. This signals that the assignments were appropriately designed. In order to enhance children’s understanding and practical use of the concept of health, it is not only important to allow flexibility in the activities and materials to best fit the children’s abilities, but also to allow adequate time for the children to be able to work with the different assignments.

### Strengths and limitations

The description of the data collection and the analysis may enhance the transparency and trustworthiness of the results, further the analysis was kept on a manifest level to minimize the risk of over-interpretation, which may increase credibility [33]. In all focus groups the children seemed to be happy and all efforts were taken to make every child feel seen, heard and respected [24]. This study contributes to the limited knowledge regarding involving children in the evaluation of health promoting and obesity preventing interventions. In addition, by maximising the variation of specific characteristics in the purposefully identified sample of children increases transferability of the findings. However, it is possible that additional perspectives of the children’s perceptions could have been captured using a larger participant sample. Schools included in this study were recruited from socioeconomically disadvantaged areas. However, among the children included in the focus groups, a high proportion of the children had parents with high education, indicating that the sample is not representative of the population in disadvantaged areas. This may limit the transferability of results when applied to families of low SEP in the wider population [37].

### Implications for practice

This study has several implications when taking the HSSP and other similar programmes to practice. When scaling-up the HSSP and other similar programmes the implementers should consider the following points, which could potentially improve the learning outcomes of the children:

- Children should be given adequate time to finish intervention activities and assignments.
- The engagement of parents and teachers should be facilitated by e.g. integration of the intervention components in the school-structure as much as possible to enhance the teacher’s possibility to give enough time and involve the parents. This could be achieved by setting up rules and expectations concerning homework assignments.
- The deliverers of information should be encouraged to tailor the lessons and the different activities to be able to reach children with different interests and abilities. Further the importance of communication through body language and being positive should be emphasized.
- The inclusion of practical activities, home assignments and the use of symbols in the material are recommended in health promotion interventions.

## Conclusions

The study shows that young children are positive towards this type of health promotion in the classroom. With a few adaptations of the intervention component, the effects on children’s learning could be enhanced. These adaptation are (1) aiming for the intervention activities such as homework to be an integrated part of the school-structure thereby engaging parents to a higher degree; (2) using flexible materials, tailored to the children’s abilities and giving children adequate time to finish activities, to enhance children’s acceptability, understanding and practical use of the concept of health; (3) making both teachers and parents aware of the importance of verbal and body language when performing intervention activities.

## Supplementary Information

**Additional file 1.** Interview guide - focus group with children in HSSP.

## Data Availability

Intervention information and materials are available on the project website (https://ki.se/gph/en-frisk-skolstart) or from the corresponding author on reasonable request.

## Abbreviations

HSSP: A Healthy School Start Plus
T2D: Type-2 diabetes
SEP: Socioeconomic position
UNCRC: The United Nations Convention on the Rights of the Child
SCT: Social Cognitive Theory
CFIR: Consolidated Framework for Implementation Research
