# Supplementary material for: Children’s experiences of participating in a school-based health promotion parental support programme – a qualitative study
Source: BMC Pediatr. 2021 May 11;21:228. doi: 10.1186/s12887-021-02694-0 (PMC8111964; doi:10.1186/s12887-021-02694-0)
Supplement: Supplementary file 1 — Additional file 1. Interview guide - focus group with children in HSSP. [file 12887_2021_2694_MOESM1_ESM.pdf]

Interview guide - focus group with children in HSSP

Presentation game

In order for us to find out a little bit more about each other, one at a time can tell your name and what your favorite dish / favorite food is. (Little teddy bear - whoever holds it can say their name and favorite dish).

<u>THE LESSONS</u>

Overall question: ***What did you think about working with food and body movement?***

FOOD:

- ***When you talked about food - what did you talk about then?*** *(Show example: show current images / things that remind)*
- ***What did you do during the lessons?***
- ***What did you think about it?***
- ***What did you find easy / good?***
- ***What did you find difficult / bad / less good / tricky?***
- ***Was it something that was too easy?***
- ***Was it something that was too difficult?***
- ***What do you others think?***
- ***Was it easy or difficult to understand when the teachers explained?***
- ***What was easy? What was difficult?***
- ***What do you think your teachers thought about it / the lessons?***
- ***How did you notice that?***
- ***What did the others in the class think about the lessons?***

PHYSICAL ACTIVITY / BODY MOVEMENT:

- ***When you talked about body movement - what did you talk about then?*** *(Show example: show current images / things that remind)*
- ***What did you do during the lessons?*** *(Put forward specific examples based on what the children mention)*
- ***What did you think about it?***

- ***What did you find easy / good?***
- ***What did you find difficult / bad / less good / tricky?***
- ***Was it something that was too easy?***
- ***Was it something that was too difficult?***
- ***What do you others think?***
- ***Was it easy or difficult to understand when the teachers explained?***
- ***What was easy? What was difficult?***
- ***What do you think the teachers thought about it / the lessons?***
- ***What did the others in the class think about the lessons?***

<u>THE WORKBOOKS</u>

Overall question: ***When you worked with food and body movement, how did you work with it at home - (if you did)?***

Do you remember this one? *(Show the 'workbook')*

- ***What did you do with it?***
- ***Who did you do these things with?*** *(Give specific examples based on what the children mention)*
- ***What did you find easy / good?***
- ***What did you find difficult / bad / less good / tricky?***
- ***When you had this - did you do something special, or different at home?*** *(Show workbook)*
- ***If filled in at home: What did mom and dad think about this?*** *(Siblings, others?)*
- ***If filled in at school: What was it like doing it with the others in the class?***
